# Precise Prostate Cancer Assessment Using IVIM-Based Parametric Estimation of Blood Diffusion from DW-MRI

**DOI:** 10.3390/bioengineering11060629

**Published:** 2024-06-19

**Authors:** Hossam Magdy Balaha, Sarah M. Ayyad, Ahmed Alksas, Mohamed Shehata, Ali Elsorougy, Mohamed Ali Badawy, Mohamed Abou El-Ghar, Ali Mahmoud, Norah Saleh Alghamdi, Mohammed Ghazal, Sohail Contractor, Ayman El-Baz

**Affiliations:** 1 Department of Bioengineering, J.B. Speed School of Engineering, University of Louisville, Louisville, KY 40292, USA; 2 Computers and Control Systems Engineering Department, Faculty of Engineering, Mansoura University, Mansoura 35516, Egypt; 3 Radiology Department, Urology and Nephrology Center, Mansoura University, Mansoura 35516, Egypt; 4 Department of Computer Sciences, College of Computer and Information Sciences, Princess Nourah bint Abdulrahman University, Riyadh 84428, Saudi Arabia; 5 Electrical, Computer, and Biomedical Engineering Depatrment, Abu Dhabi University, Abu Dhabi 59911, United Arab Emirates; 6 Department of Radiology, University of Louisville, Louisville, KY 40202, USA

**Keywords:** apparent diffusion coefficient (ADC), computer- aided diagnosis (CAD), intravoxel incoherent motion (IVIM), machine learning (ML), prostate cancer (PCa), U-Net segmentation

## Abstract

Prostate cancer is a significant health concern with high mortality rates and substantial economic impact. Early detection plays a crucial role in improving patient outcomes. This study introduces a non-invasive computer-aided diagnosis (CAD) system that leverages intravoxel incoherent motion (IVIM) parameters for the detection and diagnosis of prostate cancer (PCa). IVIM imaging enables the differentiation of water molecule diffusion within capillaries and outside vessels, offering valuable insights into tumor characteristics. The proposed approach utilizes a two-step segmentation approach through the use of three U-Net architectures for extracting tumor-containing regions of interest (ROIs) from the segmented images. The performance of the CAD system is thoroughly evaluated, considering the optimal classifier and IVIM parameters for differentiation and comparing the diagnostic value of IVIM parameters with the commonly used apparent diffusion coefficient (ADC). The results demonstrate that the combination of central zone (CZ) and peripheral zone (PZ) features with the Random Forest Classifier (RFC) yields the best performance. The CAD system achieves an accuracy of 84.08% and a balanced accuracy of 82.60%. This combination showcases high sensitivity (93.24%) and reasonable specificity (71.96%), along with good precision (81.48%) and F1 score (86.96%). These findings highlight the effectiveness of the proposed CAD system in accurately segmenting and diagnosing PCa. This study represents a significant advancement in non-invasive methods for early detection and diagnosis of PCa, showcasing the potential of IVIM parameters in combination with machine learning techniques. This developed solution has the potential to revolutionize PCa diagnosis, leading to improved patient outcomes and reduced healthcare costs.

## 1. Introduction

The prostate is a walnut-sized gland that can be found below the bladder, in front of the rectum, and behind the base of the penis. It encircles the urethra, a canal that resembles a tube that carries sperm and urine out of the penis [1]. The fundamental function of the prostate is to create seminal fluid, the substance found in semen that feeds, protects, and facilitates sperm motility. It is worth mentioning that the prostate includes three glandular regions, namely, the transition (TZ), central (CZ), and peripheral (PZ) zones, which make up around 5%, 25%, and 70% of the prostatic glandular tissue, respectively [2]. A tumor is created when normal prostate cells undergo a transformation and grow out of control. Both benign and malignant tumors are possible. Malignancy refers to the tumor’s capacity to grow and spread to other parts of the body. On the other hand, a benign tumor can form but will not grow [3]. Prostate cancer (PCa) is somewhat distinct from other malignancies. This is because many types of PCa do not easily spread to other parts of the body. In certain kinds of prostate cancer, many years or even decades may pass before any symptoms or problems are seen [1]. However, PCa is a significant health concern affecting men globally, with the second-highest incidence rate among male cancers [4].

Most cases of PCa (i.e., 70%) arise in the peripheral zone (PZ), while a smaller proportion (i.e., 20%) originate in the transitional zone (TZ) [5]. The most used imaging technique for identifying, staging, and localizing PCa is multiparametric MRI (mpMRI). However, detecting PCa that is initiated in the TZ remains challenging due to its heterogeneous appearance and the difficulty of distinguishing it from contemporaneous benign prostatic hyperplasia (BPH) [6]. The suggested starting sequence for evaluating the TZ is T2-weighted imaging (T2WI); however, its diagnostic efficacy remains poor. The diffusion-weighted imaging (DWI) sequence has emerged as a significant functional imaging technique that employs the diffusion properties of water molecules to indirectly provide a quantitative parameter called the apparent diffusion coefficient (ADC) [7]. DWI is currently the dominant sequence used to detect PZ lesions according to the PI-RADS v2.1 guidelines. However, as non-cancerous tissues such as BPH also have increased cellularity, the ADC value may be present in central zone (CZ) PCa as well. Additionally, ADC is combined with capillary microcirculation perfusion, leading to several potential false positives for PCa diagnosis [8].

With the rapid development of digital images and computing, applying artificial intelligence (AI) in medical data image processing has become a prominent research field that has achieved remarkable results [9,10]. Recently, AI, together with its machine (ML) and deep learning (DL) subfields, has made significant advances in the identification of PCa [11]. It has the potential to transform the role of clinicians, revolutionize the practice of medicine, lead to better patient care, and simplify the prediction of disease outcomes and treatment [12,13]. Consequently, numerous computer-aided diagnosis (CAD) systems have emerged as key technologies in PCa detection [14,15]. These systems employ imaging features from a fusion of MR modalities [16].

In order to distinguish between the incoherent movement of water molecules inside capillaries and the molecular diffusion outside of vessels, Le Bihan et al. [17] introduced an intravoxel incoherent motion (IVIM) in the late 1980s. While recent studies have shown encouraging findings, the best IVIM parameter to use for PCa diagnosis remains under dispute [18]. Many researchers have shown the potential role of employing the IVIM model in different cancers, e.g., brain cancer [19,20], breast cancer [21,22], rectal cancer [23], and PCa [24,25], and have demonstrated the robustness of IVIM in imaging analysis.

The current study aims to refine the use of IVIM imaging in the diagnosis of PCa using ML techniques. Further, it seeks to extend the use of IVIM parameters to both the CZ and PZ of the prostate for better diagnostic efficacy. Additionally, a two-stage segmentation is combined with IVIM parameter estimation to accurately localize lesions and preprocess data. The objective of the study is to analyze the diffusion parameters of the cases and evaluate the performance of a non-invasive IVIM-based CAD system for classifying regions of interest (ROIs) as either PCa or benign prostatic hyperplasia. The study also determines the best classifier and IVIM parameters for differentiation and compares the diagnostic value of IVIM parameters with that of ADC. This paper can be considered a significant contribution to the development of non-invasive methods for early detection and diagnosis of PCa, as it uses IVIM parameters in both the central and peripheral zones for the first time. The following points provide a summary of the study’s contributions:

- Development of a non-invasive CAD system: This paper introduces a novel cascaded framework for PCa detection and segmentation using diffusion-weighted magnetic resonance imaging (DW-MRI). The framework consists of several stages, including image acquisition, prostate segmentation, lesion segmentation, IVIM parameter extraction, dataset normalization, learning and optimization, and model evaluation. This framework provides a systematic approach for accurate and efficient detection and segmentation of PCa lesions.
- Utilization of U-net architecture: This paper employs a U-net architecture in two different flavors for prostate and lesion segmentation. By utilizing a U-net design, the framework effectively locates and separates PCa lesions within the prostate region. This contribution highlights the application of state-of-the-art deep learning techniques in the field of PCa detection.
- Extraction of IVIM parameters: This paper focuses on extracting IVIM parameters from DW-MRI images. The IVIM model describes the diffusion of water molecules in tissue using molecular diffusion and microcirculation processes. By extracting IVIM parameters, the framework captures valuable information about tissue microstructure, which can aid in the detection and diagnosis of PCa. This contribution highlights the integration of advanced diffusion modeling techniques in the proposed framework.
- Contribution to non-invasive methods for PCa detection: By utilizing IVIM parameters in both the CZ and PZ, this study makes a significant contribution to the development of non-invasive methods for early detection and diagnosis of PCa. The findings have the potential to improve current practices and enhance the overall management of PCa patients.

The rest of this paper is organized as follows: Section 2 provides an overview of related studies in the field; Section 3 describes the research materials; Section 4 describes the methodology; Section 5 describes the experimental setup and results; Section 6 presents an overall discussion of the study; and Section 7 concludes the paper and offers insights into future work.

## 2. Related Studies

Numerous published studies have delved into the role of the intravoxel incoherent motion (IVIM) parameter in the grading of various tumors. The concept of capturing the distinction between tissue perfusion and diffusion effects within the IVIM model was initially introduced by Le Bihan et al. [17]. This approach offered a more dependable methodology for characterizing microcirculation-induced signal attenuation than the simplistic mono-exponential model. Recent investigations have examined IVIM’s potential in different domains, including differentiation between spinal metastasis and tuberculous spondylitis [26] and assessment of glioma malignancy and isocitrate dehydrogenase 1 (IDH-1) gene type [27]. However, Le Bihan [28] proposed that perfusion could be accurately estimated from diffusion-weighted imaging (DWI) alone, obviating the need for contrast agent-based magnetic resonance (MR) imaging techniques such as dynamic contrast-enhanced (DCE) imaging.

Multiple investigations have leveraged the IVIM model in the grading of prostate cancer (PCa) tumors. Research has demonstrated that the *D* parameter of the IVIM model outperforms other parameters in Gleason score (GS) grading of PCa tumors [29]. Nevertheless, other studies have highlighted the relevance of both the *D* parameter and apparent diffusion coefficient (ADC) to cell density in PCa and the potential for deriving treatment outcome insights from changes in IVIM parameters during treatment [30,31].

Meng et al. [32] aimed to evaluate the predictive utility of IVIM for positive surgical margins (PSMs) and Gleason score (GS) upgrading in PCa patients undergoing radical prostatectomy (RP). In a retrospective analysis of 106 eligible PCa patients, pelvic multiparametric Magnetic Resonance Imaging (mpMRI) was employed. IVIM parameters were derived using postprocessing software and LR models were applied to identify predictive risk factors for PSMs and GS upgrading. The results indicated that the combination of IVIM and clinical factors enhanced the prediction of PSMs, albeit with limited improvements in predicting GS upgrading mainly involving enhanced sensitivity. The study concluded that IVIM displays effective predictive capabilities for PSMs and GS upgrading, potentially enhancing clinical diagnosis and treatment in conjunction with clinical factors.

Additionally, Hu et al. [33] aimed to investigate the predictive potential of 3D amide proton transfer-weighted (APTw) and IVIM imaging alongside routine diffusion-weighted imaging (DWI) for detecting bone metastasis (BM) in PCa patients. Their retrospective analysis encompassed 39 PCa patients grouped into BM-negative and BM-positive categories. MR examination incorporated APTw, DWI, and IVIM imaging, with IVIM data fitted using single-exponential (IVIMmono) and double-exponential (IVIMbi) models. IVIM demonstrated superior performance compared to APTw and DWI, with the single-exponential model surpassing the double-exponential model. Combining the APTw and IVIMmono further enhanced diagnostic accuracy. Overall, both APTw and IVIM imaging exhibited effectiveness in predicting PCa bone metastasis, with IVIM showing greater promise and potential for improved diagnostics when combined with APTw.

Sen et al. [34] studied whether quantitative diffusion MRI (i.e., ADC, IVIM, DKI, VERDICT) can differentiate various tissue types (i.e., false positives, true positives, normal tissue). They examined 38 patients with mp-MRI and VERDICT MRI after transperineal biopsies, categorizing them as 19 significant cancers and 19 atrophy/inflammation/high-grade prostatic intraepithelial neoplasias. Clinical ADC values and deep learning were used to analyze IVIM, DKI, and VERDICT. Significant distinctions (p<0.05) between true and false positive lesions were found in ADC, IVIM perfusion fraction (*f*), diffusivity (*D*), DKI diffusivity ($D_{K}$), kurtosis (*K*), VERDICT intracellular volume fraction ($f_{IC}$), extracellular–extravascular volume fraction ($f_{EES}$), and VERDICT diffusivity ($d_{EES}$) (p<0.0001). False positive lesions differed significantly from normal tissue in VERDICT intracellular volume fraction ($f_{IC}$) (p=0.004) and IVIM diffusivity (*D*). Quantitative diffusion MRI techniques (ADC, IVIM, DKI, VERDICT) effectively differentiated false positive lesions and cancer, potentially reducing unnecessary biopsies. A limitation of this study was its relatively small patient group.

In addition, Chen et al. [24] assessed the relationship between IVIM characteristics and PCa severity. The IVIM technique was utilized with five different b-values (i.e., b0, b188, b375, b563, b750) to acquire data. Several parameters were computed using histogram analysis, including ADC, pseudo-diffusion volume fraction ($f_{p}$), pure tissue diffusion ($D_{s}$), hypoxia score ($HS_{DWI}$), hypoxic fraction ($HF_{DWI}$), and relative oxygen saturation ($rOS_{DWI}$). The authors also explored the discriminative power of IVIM-derived hypoxia parameters for distinguishing low-grade PCa (grade ≤2) from high-grade PCa (grade ≥3). They employed cross-validated SVM classification and compared the results with univariate ROC analysis to explore the potential of using IVIM-derived hypoxia for stratifying the risk of PZ PCa. During cross-validation, their prediction model incorporating IVIM-derived hypoxia achieved an AUC ranging from 0.749 to 0.786. Although they achieved promising results for the training data, only moderate results were attained for the testing data.

Using image features from mpMRI, Li et al. [35] performed a study to assess the efficiency of SVM classification in classifying the GS of PCa within the central gland (CG). Through a correlation between radiological and pathological data, they identified 152 ROIs within the CG that were cancerous. From the mpMRI and histogram analysis, they derived eleven parameters, including mean, median, the 10th percentile, skewness, and kurtosis for each parameter (IVIM and conventional ADC parameters). In total, they calculated 55 variables, which were used as inputs for the SVM model. The model was developed using a 10-fold cross-validation approach and was further validated using two distinct datasets. Their findings revealed that SVM classification based on image characteristics derived from mpMRI consistently achieved an accurate classification of the Gleason Score of prostate cancer within the central gland.

To demonstrate the effectiveness of hierarchical clustering (HC) as a method for diagnosing PCa, Akamine et al. [36] constructed an HC model using mp-MRI, which includes diffusion kurtosis imaging, IVIM, and dynamic contrast-enhanced MRI data. The HC model’s optimization involved evaluating various combinations of dissimilarity and linkage methods. The model’s quality was confirmed through internal validation methods. The optimal HC model achieved an accuracy of 96.3% for discriminating between tumor and normal tissue in the PZ and 97.8% in the TZ. According to the study’s findings, hierarchical clustering demonstrated the ability to effectively differentiate between prostate cancer and normal tissue. However, this research had limitations, notably the small patient sample size, the use of only eight input parameters from DWI, and the use of permeability data to construct the HC model.

Furthermore, Alkadi et al. [37] investigated the classification potential of various b-values in diffusion-weighted MRI (DW-MRI) for detecting PCa. Analyzing DW-MR data collected from 20 patients (classified as nine and eleven malignant and benign cases, respectively, with varying Gleason scores), they compared different parametric maps generated through diverse b-value combinations and fitting models. They developed a machine learning-based computer-assisted diagnosis system utilizing the most effective maps, achieving an impressive 90% accuracy and an AUC of 0.978 for distinguishing early-stage PCa. This study highlights that incorporating low b-values and optimizing b-value distribution can enhance the accuracy of PCa diagnosis using DWI. However, the study encountered limitations, such as challenges in collecting sixteen different b-values from hospitals and a relatively small patient cohort.

Moreover, Merisaari et al. [38] investigated different fitting approaches for IVIM imaging in PCa regarding their ability to predict GS and their repeatability. They conducted two DWI scans on 81 PCa patients using fourteen different b-values spanning 0 to 500 s/mm^2^. They applied five IVIM fitting techniques, along with monoexponential, kurtosis, and stretched exponential models, to fit the mean signal intensities of specific ROIs. The tumors were categorized into three groups (3+3, 3+4, >3+4), then machine learning algorithms were employed to evaluate different parameter combinations. Their results showed that the monoexponential model outperformed IVIM when assessed using the Akaike information criteria (AIC). Different combinations of parameters did not exceed the performance of the monoexponential model.

Liu et al. [39] introduced a two-phase approach for the complete automation of prostate lesion detection and classification. This approach involved utilizing input sequences of T2-weighted images, ADC maps, and diffusion-weighted images with high b-values. In the initial phase, they employed a Mask R-CNN model to autonomously segment prostate structures. In the subsequent phase, a weakly supervised deep neural network was developed to both identify and categorize lesions in a single operation. To assess the accuracy of their approach, they validated it on two distinct datasets, one from the PROSTATEx Challenge and the other gathered from their local cohort. This method demonstrated impressive performance, with respective AUC values of 0.912 and 0.882 on the two datasets.

Table 1 provides a comprehensive overview of various studies investigating the role of IVIM parameters in the detection and characterization of PCa. These studies, spanning from the late 1980s to the present, utilize diverse imaging modalities such as DW-MRI and mpMRI. Notably, Le Bihan et al. [17] introduced the concept of IVIM to distinguish tissue perfusion and diffusion effects, suggesting the possibility of accurately estimating perfusion from DWI alone. Subsequent studies explored IVIM’s potential in differentiating spinal metastasis, assessing glioma malignancy, and evaluating PCa tumors. Researchers have investigated the predictive utility of IVIM for positive surgical margins and Gleason score upgrading, leading to enhanced diagnostic capabilities when combined with clinical factors. Additionally, ML techniques have been employed to classify PCa severity and automate lesion detection. The table below underscores the evolving landscape of PCa diagnosis, showcasing advancements in imaging technologies and analytical methodologies that ultimately contribute to improved understanding and diagnosis of this prevalent malignancy.

It is worth noting that while a considerable number of previous investigations have explored the utility of IVIM parameters and ADC in PCa detection, only a limited number have delved into the potential of ML techniques. Consequently, there remains a notable gap in our understanding. However, the proposed approach aims to bridge this deficiency by presenting a novel non-invasive CAD system that harnesses IVIM parameters for the detection and diagnosis of PCa. This innovative strategy holds the promise of addressing current limitations and contributing to enhanced and more accurate PCa diagnosis through the integration of advanced ML methodologies.

## 3. Materials

Study design and ethical considerations: This study was conducted in accordance with the principles outlined in the Declaration of Helsinki and received ethical approval from the institutional review board (IRB). Written informed consent was obtained from all participating patients prior to their inclusion in the study, ensuring adherence to ethical guidelines and regulations.

Patient selection and characteristics: Between 2019 and 2021, a cohort of 80 individuals exhibiting clinical indications of potential prostate cancer underwent comprehensive evaluation. Eligibility criteria included a positive digital rectal examination and elevated serum prostate-specific antigen (PSA) levels (>4 ng/mL). The participants had an average age of 66 years (age range: 48–82, standard deviation: 7.119) and were categorized into two groups: 37 patients were diagnosed with prostatic carcinomas, while 43 patients were diagnosed with benign prostatic hyperplasia.

Imaging techniques: All magnetic resonance imaging (MRI) scans were performed on a 3 Tesla MRI scanner (Phillips, Ingenia 3T, The Best, Netherlands) equipped with a phased-array body coil. The multiparametric MRI (mp-MRI) protocol encompassed the assessment of various parameters, focusing on the prostate’s central and peripheral zones. Lesions in both zones were included.

The mp-MRI protocol comprised the sequences below. Our comprehensive imaging protocol facilitated a detailed evaluation of prostate lesions and provided essential data for subsequent analyses.

- T2-weighted Turbo Spin Echo (TSE) imaging: Standard axial, coronal, and sagittal T2-weighted TSE sequences were acquired. The imaging parameters were as follows: repetition time (TR)/echo time (TE) = 3672/110 ms, slice thickness/gap =3/0.3 mm, field of view (FOV) =16 cm, matrix =320×176.
- Diffusion-Weighted Imaging (DWI): Axial DWI scans were acquired using a single-shot echo-planar imaging technique. The imaging parameters were: TR/TE =4735/88 ms, slice thickness/gap =3.5/0.1 mm, FOV =200×178 mm, matrix =68×59, SENSE factor =2. A series of b-values (i.e., 0, 100, 200, 300, 400, 500, 600, 700, and 1400 s/mm^2^) were employed to probe diffusion characteristics. The acquisition time for the DWI sequence was approximately 7 min and 8 s.

Data collection and analysis: Detailed patient information was collected, including age, b-values, T2 measurements, PSA levels, follow-up PSA, responses to questionnaires, number of biopsy sectors, Gleason Score, grade, diagnosis, and treatment outcomes. Data obtained from the mp-MRI protocol and patient information were subjected to rigorous analysis, including image processing and quantitative assessments. However, some patients did not record certain information, such as PSA level and follow-up PSA.

Statistical analysis: Statistical analyses were performed to explore the relationships between imaging parameters, patient characteristics, and clinical findings.

## 4. Methodology

**Figure 1:**
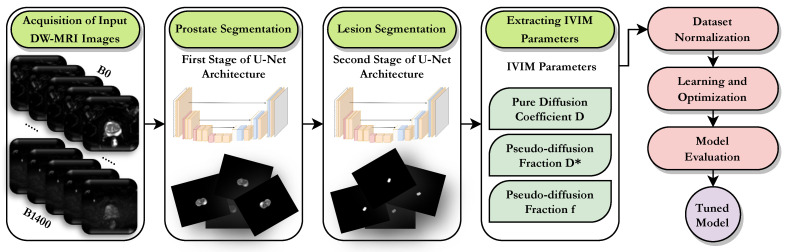
Stages of the proposed IVIM-based CAD system pipeline: acquisition of input DW-MRI images, prostate segmentation, lesion segmentation, extracting IVIM parameters, dataset normalization, learning and optimization, model evaluation.

The proposed framework for prostate cancer detection and segmentation using diffusion-weighted magnetic resonance imaging (DW-MRI), presented in Figure 1, consists of several cascaded stages. The first stage involves the acquisition of input DW-MRI images, which typically range from b=0 to b=1400. The b-value indicates both the strength and timing of the gradients utilized in producing diffusion-weighted images. The diffusion characteristics of water molecules within tissues are captured by the DW-MRI images, which may be used to identify anomalies such as prostate cancer. The prostate is segmented in the second stage of the framework, while the lesion is segmented in the third stage. Both of these stages utilize a U-net architecture, with two different flavors. U-net designs are frequently employed for image segmentation applications, being applied in this situation to locate and separate prostate cancer lesions inside the prostate region. The fourth stage of the framework involves extracting IVIM parameters from the DW-MRI images. The IVIM model describes the diffusion of water molecules in tissue as a combination of two processes, namely, molecular diffusion and microcirculation. IVIM parameters can provide valuable information about tissue microstructure, which can be used to detect and diagnose prostate cancer.

The fifth stage of the framework involves dataset normalization. The normalization process involves scaling and centering the input DW-MRI images and the extracted IVIM parameters to ensure that they are on the same scale. This step is important for improving the performance of the subsequent learning and optimization stages. The sixth stage of the framework involves learning and optimization using an ML algorithm. The ML algorithm is trained on the normalized DW-MRI images and IVIM parameters to develop a predictive model that can accurately detect and segment prostate cancer lesions. The seventh and final stage of the framework involves model evaluation. The tuned model is evaluated on a testing dataset to assess its performance in detecting and segmenting prostate cancer lesions. Standard metrics, including balanced accuracy, specificity, sensitivity, and Dice coefficient, are used to assess the performance of the presented models.

### 4.1. Prostate and Lesion Segmentation

The proposed framework consists of two stages for automatic segmentation, with the first stage focused on prostate segmentation and the second stage on lesion segmentation. To enhance segmentation accuracy, a pipeline consisting of a deeply supervised 2D U-Net and two different flavors is utilized in this stage. These variants are U-Net, Attention U-Net, and V-Net. U-Net is a popular convolutional neural network (CNN) architecture for image segmentation tasks. It was introduced in a 2015 paper by Ronneberger et al. [40], and has since become a widely-used approach for biomedical image segmentation. The key feature of U-Net is its U-shaped architecture, which consists of an encoder network that progressively reduces the spatial resolution of the input image, followed by a decoder network that upsamples the feature maps back to the original resolution. The encoder and decoder networks are connected by skip connections, which allow the decoder to access low-level features from the encoder. The U-net architecture has been shown to be effective for various medical imaging applications, including segmentation of the brain, lungs, and liver. Attention U-Net is a variation of the U-net architecture that incorporates the attention mechanism concept [41].

Attention mechanisms allow the network to selectively focus on the most informative regions of the input image rather than processing the entire image equally. This can be particularly useful for medical imaging tasks, where the regions of interest may be small and difficult to distinguish from the background. The attention mechanism is typically implemented using a gating mechanism that learns to weigh the importance of each feature map. Attention U-Net has been shown to outperform the standard U-Net on several medical image segmentation tasks. V-Net is another CNN architecture used for volumetric medical image segmentation tasks. It was introduced in a 2016 paper by Milletari et al. [42], and is similar to U-Net in that it uses an encoder–decoder architecture with skip connections. However, V-Net also incorporates 3D convolutions, which allow it to process volumetric data directly without the need for additional preprocessing steps. V-Net also includes residual connections, which can help to mitigate the vanishing gradient problem that can occur in very deep networks. The V-Net architecture has been shown to achieve state-of-the-art results on several medical image segmentation tasks, including segmentation of the brain, liver, and kidneys [43].

Table 2 presents the hyperparameters used for each segmentation model in the current study. Each model has several parameters with specific values. The parameter “Filter Num” represents the number of filters in each convolutional block. The parameters “Stack Num Down” and “Stack Num Up” determine the number of convolutional blocks in the down-sampling and up-sampling paths, respectively. The “Activation” parameter specifies the activation function used in the convolutional layers. The “Atten Activation” parameter, which is specific to Attention U-Net, specifies the activation function used in the attention layers, while the attention parameter specifies the type of attention mechanism used. The “Batch Norm” parameter is a Boolean indicating whether to use batch normalization, while the “Backbone” parameter specifies the pretrained model used as a backbone, which is only applicable for U-Net and Attention U-Net. The “Pool” and “Unpool” parameters are Booleans that indicate whether to use pooling or unpooling, respectively. The “Freeze Backbone” and “Freeze Batch Norm” parameters are Booleans that indicate whether to freeze the weights of the backbone and batch normalization layers, respectively. The “Weights” parameter, which is specific to Attention U-Net, specifies the weight initialization method. The V-Net model has two additional parameters: “# Res Ini”, which specifies the number of residual blocks in the initial convolutional block, and “# Res Max”, which specifies the maximum number of residual blocks in the convolutional blocks.

**Figure 2:**
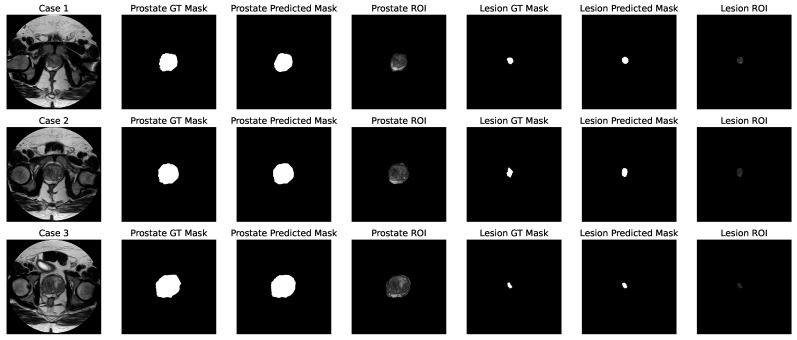
Visualization of three cases, each presented in a row. From left to right, the columns represent the entire scan, the prostate GT mask, the prostate predicted mask, the prostate ROI, the lesion GT mask, the lesion predicted mask, and the lesion ROI.

Figure 2 illustrates three cases, with each case displayed in a separate row. From left to right, the columns represent the entire scan, the prostate GT mask, the prostate predicted mask, the prostate ROI, the lesion GT mask, the lesion predicted mask, and the lesion ROI.

### 4.2. DWI Analysis Using IVIM Fitting

The IVIM model is a mathematical representation that describes the signal attenuation in DWI by considering both diffusion and perfusion effects. It assumes that the measured signal intensity S(b) is a combination of two components: (1) the diffusion-related component, and (2) the perfusion-related component. IVIM diffusion imaging can be considered advantageous over other diffusion models due to its ability to separate and quantify perfusion and diffusion effects within tissues. IVIM provides valuable information about both microcirculation and pure diffusion, allowing for more comprehensive characterization of tissue properties. Unlike other models that primarily focus on diffusion, IVIM incorporates perfusion-related parameters such as blood flow and capillary permeability, which are essential in assessing tissue viability, disease progression, and treatment response. This unique feature of IVIM makes it particularly useful in areas such as oncology, where the evaluation of tumor vascularity and microstructure is critical for diagnosis and therapeutic monitoring [44].

The Intravoxel Incoherent Motion (IVIM) model offers a mathematical framework for elucidating how the DWI signal diminishes, incorporating both diffusion and perfusion phenomena. It posits that the observed signal intensity S(b) is a blend of two primary components: (1) diffusion-driven attenuation, and (2) perfusion-induced effects. This model proves advantageous in that it disentangles and quantifies these distinct contributions within tissue to provide a nuanced understanding of microcirculation and pure diffusion dynamics. Unlike conventional diffusion models, IVIM integrates perfusion-related metrics such as blood flow and capillary permeability, which are crucial for assessing tissue vitality, disease evolution, and treatment efficacy. This nuanced approach finds particular relevance in oncology, facilitating the precise evaluation of tumor vascularity and microstructure that is pivotal for diagnosis and therapy tracking [44]. Equation (1) can be used to explain the IVIM model [17,37]. The IVIM model equation can be interpreted as a weighted sum of two exponential decay terms. The first term, $\left(1-f\right)\times e^{-b\times D}$, represents the signal attenuation due to pure molecular diffusion. It decays exponentially with the increase in the diffusion weighting factor *b*. The second term, $f\times e^{-b\times D^{*}}$, accounts for the perfusion-related effects. It also decays exponentially with *b*, but with a different rate determined by $D^{*}$. By fitting the IVIM model equation to the acquired DWI data, the parameters S(0), *f*, *D*, and $D^{*}$ can be estimated. These parameter values provide quantitative information about tissue diffusion and perfusion characteristics. The perfusion fraction (*f*) and diffusion coefficients (*D* and $D^{*}$) can offer valuable insights into tissue microstructure, blood flow, and treatment response in various clinical applications [28,45].

(1) $$S\left(b\right)=S(0)\times \left(f\times e^{-b\times D^{*}}+\left(1-f\right)\times e^{-b\times D}\right)$$

Below, we break down Equation (1) with reference to the physical phenomena of diffusion and perfusion:

- S(b) represents the signal intensity measured with diffusion weighting *b*. This signal is influenced by both diffusion and perfusion effects.
- S(0) is the baseline signal intensity obtained without any diffusion weighting (b=0). It serves as a reference point for the signal intensity.
- *f* is the perfusion fraction, indicating the proportion of the signal arising from perfusion effects. It quantifies the contribution of perfusion to the total signal.
- *D* is the diffusion coefficient, characterizing the pure molecular diffusion of water molecules within tissue. It represents the diffusion-related component of signal attenuation.
- $D^{*}$ is the pseudo-diffusion coefficient, which accounts for microcirculation-related effects such as blood flow and capillary perfusion. It represents the perfusion-related component of signal attenuation.

Next, we discuss the physical interpretations of these parameters:

- S(0): This term represents the baseline signal intensity, which is unaffected by diffusion. In tissues with high cellularity or restricted diffusion, S(0) tends to be higher, reflecting reduced water mobility.
- *f*: The perfusion fraction quantifies the fraction of the signal contributed by perfusion effects. In tissues with high vascularity or blood flow, *f* is higher, indicating a larger contribution of perfusion to the total signal.
- *D*: The diffusion coefficient represents the rate of pure molecular diffusion of water molecules within tissue. Tissues with high cell density or structural barriers exhibit lower *D* values, indicating restricted diffusion.
- $D^{*}$: The pseudo-diffusion coefficient reflects microcirculation-related effects such as blood flow and capillary perfusion. Elevated $D^{*}$ values suggest increased microvascular perfusion, as is often observed in highly vascularized tissues or regions with increased blood flow.

**Figure 3:**
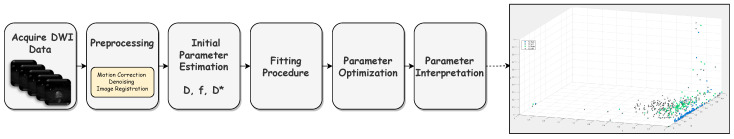
Graphical presentation of Algorithm 1; the rightmost plot represents the PZ and CZ regions for the left and right prostates using the gathered IVIM parameters.

Algorithm 1 presents the followed major steps in calculating the IVIM parameters. Figure 3 reflects the algorithm in a graphical manner.
**Algorithm 1:** Major steps followed to calculate the IVIM parameters**1** **Step 1: Acquire DWI Data**: A series of DWI images with different diffusion weighting factors (*b*-values) are acquired. The signal intensities (S(b)) are obtained for each *b*-value.**2** **Step 2: Preprocessing**: Before fitting the IVIM model, some preprocessing steps can be utilized. The current study utilized motion correction, denoising, and image registration to ensure the quality and alignment of the DWI data.**3** **Step 3: Initial Parameter Estimation**: Initial estimates of the IVIM parameters $\left(S\left(0\right),f,D,D^{*}\right)$ are required to initialize the fitting procedure. These initial estimates can be obtained through various methods, such as region-of-interest (ROI) analysis, manual selection, or prior knowledge from literature.**4** **Step 4: Fitting Procedure**: The fitting procedure involves minimizing the difference between the measured DWI data (S(b)) and the model-predicted signal intensities based on the IVIM equation. Nonlinear least squares fitting is commonly employed, which aims to minimize the sum of squared residuals. The fitting algorithm iteratively adjusts the parameter values to minimize the difference between the model and the measured data.**5** **Step 5: Parameter Optimization**: The fitting procedure continues until convergence, where the optimal parameter values are obtained. Convergence is typically determined based on a predefined criterion, such as reaching a specific tolerance level or a maximum number of iterations.**6** **Step 6: Parameter Interpretation**: Once the optimal parameter values are determined, they can be interpreted to gain different insights into tissue diffusion and perfusion characteristics. For example, the perfusion fraction (f) provides information about the contribution of perfusion to the overall signal, while the diffusion coefficients (*D* and $D^{*}$) quantify the diffusion properties of tissue and microcirculation-related effects.

When performing nonlinear least squares fitting, the objective is to minimize the difference/residuals ((R)) between the measured DWI data ($\left(S_{measured}\left(b\right)\right)$) and the model-predicted signal intensities ($S_{prediction}\left(b\right)$). This is presented in Equation (2), where $S_{prediction}\left(b\right)$ represents the model-predicted signal intensity, which can be calculated using Equation (3). The Model in this equation refers to the trained machine learning classifier (see Section 4.4), while $\left(S_{measured}\left(b\right)\right)$ is calculated using Equation (1).

The objective of this fitting procedure is to determine the IVIM parameter values $\left(S\left(0\right),f,D,D^{*}\right)$ that minimize the sum of squared residuals. As mentioned, the fitting algorithm is run iteratively to adjust the parameter values with the aim of achieving this minimization. The sum of the squares’ residuals (SSR) is presented in Equation (4), where *N* reflects the number of data points. The Levenberg–Marquardt algorithm is utilized to minimize the (SSR) value [46].

(2) $$R=S_{measured}\left(b\right)-S_{prediction}\left(b\right)$$

(3) $$S_{prediction}\left(b\right)=Model\left[(S\left(0\right),f,D,D^{*})\right]$$

(4) $$SSR=\sum_{i=1}^{N}R_{i}^{2}=\sum_{i=1}^{N}{\left(S_{measured}\left(b\right)-S_{prediction}\left(b\right)\right)}^{2}$$

Here, we are aiming to compare the effectiveness of the bi-exponential model’s IVIM parameters with the mono-exponential ADC model in distinguishing between BPH and PCa. The conventional ADC calculation was performed using Equation (5). As mentioned earlier, the term $\frac{S(b)}{S(0)}$ indicates the signal loss resulting from the movement of water in the blood vessels at a specific b-value of the DWI scan. To reduce the impact of noise and maintain the continuity of ADC, a continuity correction technique, also known as smoothing, was used.

(5) $$ADC=-\frac{ln\left(\frac{S(b)}{S(0)}\right)}{b}$$

### 4.3. Dataset Normalization

Data normalization is an essential step in the data preparation process for any research paper. It refers to the process of transforming data into a standard format that allows for easier analysis and comparison across different variables and samples [47]. Data normalization is important for several reasons. First, normalizing data reduces the impact of differences in measurement units or scales, which can lead to bias in the analysis. By ensuring that all variables are on a common scale, no variable can unduly influence the results. Second, normalizing data allows data from different sources or different variables to be compared meaningfully. This is particularly important in cases where the same variable is measured using different units of measurement. Third, normalizing the data can help to identify and correct errors or outliers in the data. This can improve the accuracy of the analysis and increase the validity of the research findings. Finally, normalized data are often easier to analyze and interpret than raw data. By ensuring that the data are in a standard format, it is easier to apply statistical techniques and perform meaningful comparisons [48].

### 4.4. Classification of Lesions

In this study, the performance of various machine learning classifiers was evaluated for the task of lesion classification. Lesion classification is a critical step in medical image analysis, as it plays a crucial role in assisting medical professionals in diagnosing and treating diseases [49]. Multi-Layer Perceptron Classifiers (MLP) is a type of feedforward neural network that consists of multiple layers of nodes (neurons) and is widely used for classification tasks. It is capable of learning complex patterns and relationships in the data. Decision Tree (DT) is a simple yet effective classifier that uses a hierarchical structure of decision rules to classify instances. It partitions the feature space into distinct regions based on their feature values, then makes predictions based on the majority class in each region. K-Nearest Neighbors (KNN) is a nonparametric classifier that assigns labels to instances based on the labels of their nearest neighbors in the feature space. It classifies an instance by a majority vote of its *k* nearest neighbors.

Extra Tree Classifier (ERTC) is an ensemble learning method that combines multiple decision trees. It introduces randomness into the process of constructing the decision tree by selecting random thresholds for splitting and choosing random subsets of features [50]. AdaBoost Classifier (ADA) is an ensemble learning method that combines multiple weak classifiers to create a strong classifier. It assigns weights to instances based on their classification errors and adjusts the weights iteratively to focus on difficult instances. Random Forest Classifier (RFC) is an ensemble learning method that combines multiple decision trees to make predictions. It introduces randomness by using bootstrap samples of the training data and randomly selecting a subset of features at each split. Extra Trees Classifier (ETC) is another ensemble learning method that combines multiple decision trees. Similar to RFC, it introduces randomness by using bootstrap samples and random feature subsets. Gradient Boosting Classifier (GB) is an ensemble learning method that builds a strong classifier by iteratively adding weak classifiers. It optimizes a loss function by adjusting the weights of the weak classifiers based on their individual performance [51].

Histogram-Based Gradient Boosting Tree Classifier (HGB) is an extension of gradient boosting that utilizes histograms to speed up computation and improve memory efficiency. It constructs histograms of the features and uses them to find the best splits during the boosting process. XGBoost Classifier (XGB) is an optimized implementation of gradient boosting that utilizes a tree-based learning algorithm. It incorporates regularization techniques and parallel computing to enhance performance and accuracy. LightGBM Classifier (LGBM) is another gradient boosting framework that aims to achieve high efficiency and accuracy. It uses a novel tree-based learning algorithm and employs a leaf-wise strategy for growing the trees. These classifiers were chosen based on their effectiveness and popularity in the field of machine learning and their suitability for the task of lesion classification. Each classifier has its own strengths and weaknesses, and their performance can vary depending on the specific dataset and application. By evaluating and comparing the performance of these classifiers, this study aims to identify the most effective and accurate classifier for lesion classification. The evaluation is based on numerous performance metrics, including accuracy, sensitivity, specificity, F1-score, precision, and area under the receiver operating characteristic curve (AUC-ROC), as described below [52].

### 4.5. Performance Measures

To analyze the effectiveness of various modalities and classifiers for the assessment and segmentation task, multiple performance metrics were used. These metrics provide important information on the efficiency and dependability of the suggested methods. The accuracy of a classifier’s predictions is a metric that indicates how generally accurate it is. It is determined by dividing the number of correctly categorized samples by the total number of samples [53,54]. Higher accuracy reflects the classifier’s improved ability to properly classify both positive and negative cases. Sensitivity, commonly referred to as the true positive rate or recall, measures how well a classifier can recognize positive cases. It is computed as the ratio of true positive samples to the total of true positive and false negative samples [55]. An important factor in reducing false negatives is the capacity to detect actual positive samples, which is indicated by greater sensitivity scores. The classifier’s specificity is measured by how well it can recognize negative occurrences. The ratio of true negative samples to the total of true negative and false positive samples is used to compute it. In order to reduce false positives, it is crucial to have a higher specificity value, which indicates a greater capacity to detect actual negative samples [56].

The percentage of accurately anticipated positive cases out of all expected positive instances is known as the precision. The ratio of true positive samples to the total number of true positive and false positive samples is used to compute it [57]. A greater precision value denotes a reduced rate of false positives. The harmonic mean of memory and sensitivity is known as the F1 Score [58]. When the dataset is unbalanced, this metric can offer greater balance between precision and sensitivity. A higher value denotes a better balance between precision and sensitivity. The F1 score takes a value between 0 and 1. The Receiver Operating Characteristic (ROC) Curve is a graphical depiction of the classifier’s performance at different discrimination thresholds. It plots the true positive rate (sensitivity) against the false positive rate (1-specificity) for different threshold values. The area under the ROC curve (AUC) provides a measure of the classifier’s discrimination ability, with a higher AUC indicating better performance [59].

Balanced Accuracy calculates the average of sensitivity and specificity. It provides an overall measure of the classifier’s performance that takes into account both true positive and true negative rates [60]. It is particularly useful when the dataset is imbalanced, as it accounts for uneven distribution of the classes. The Dice coefficient quantifies the similarity between the predicted and ground truth segmentations by calculating the ratio of the overlap between the two to the total number of pixels in both. It provides a balanced measure that rewards both precision and recall, making it suitable for evaluating the segmentation of irregularly shaped lesions. Additionally, the IoU measures the overlap between the predicted and ground truth segmentations by calculating the intersection divided by the union of the two regions [60,61].

**Figure 4:**
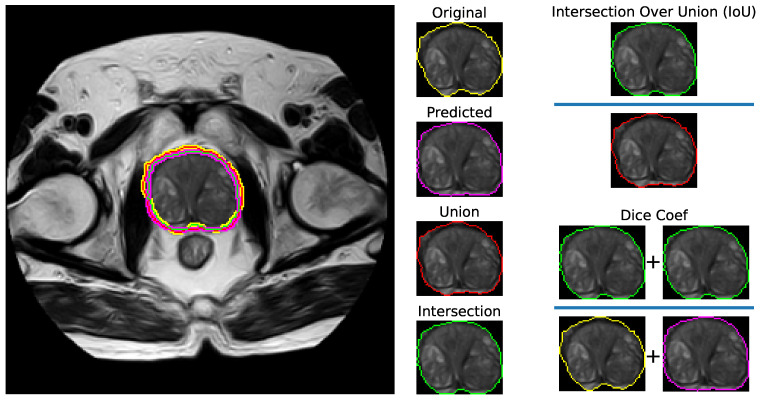
Visualization of the Dice coefficient and intersection over union metrics for segmentation. The color scheme for the contours is as follows: yellow for the original, purple for the prediction, red for the union, and green for the intersection.

**Figure 5:**
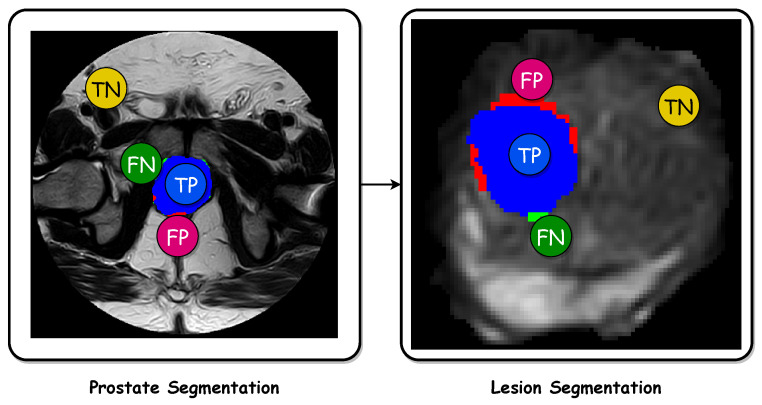
Images displaying visual samples of the segmented sections along with the true positives (TP), true negatives (TN), false positives (FP), and false negatives (FN). The two subplots on the left refer to the prostate segmentation, while the four subplots on the right refer to the lesion segmentation plotted on the whole scan and prostate, respectively. In the images, green indicates FN, blue represents TP, orange indicates TN, and red signifies FP.

Figure 4 shows a visualization of the Dice coefficient and IoU metrics regarding the segmentation. Figure 5 displays visual examples of the segmented sections for the prostate and lesion accompanied by the corresponding true positives (TP), false positives (FP), and false negatives (FN). The two subplots on the left depict the prostate segmentation, while the four subplots on the right show the lesion segmentation plotted on the whole scan and prostate, respectively. In the images, green indicates FN, blue represents TP, orange indicates TN, and red signifies FP.

### 4.6. Pseudocode for the Overall Framework

Algorithm 2 outlines the suggested detection and segmentation framework for PCa using DW-MRI. The algorithm encompasses a multistage process, beginning with the acquisition of DW-MRI images with varying diffusion weighting factors (*b*-values). Subsequently, preprocessing steps, including motion correction, denoising, and image registration, are applied to ensure data quality. The algorithm then proceeds to prostate and lesion segmentation using U-net architecture variants, namely, Attention U-Net and V-Net, to identify and separate cancerous regions. Following this, the IVIM model is employed to extract essential parameters such as S(0), perfusion fraction (*f*), diffusion coefficient (*D*), and pseudo-diffusion coefficient ($D^{*}$). The dataset normalization step scales and centers the DW-MRI images and IVIM parameters to a common scale. Subsequently, ML algorithms are trained on the normalized data for cancer detection and segmentation. The final stage involves model evaluation on a testing dataset, utilizing performance metrics such as balanced accuracy, specificity, sensitivity, and Dice coefficient. This pseudocode encapsulates a systematic approach to prostate cancer analysis, integrating imaging, mathematical modeling, and machine learning for enhanced diagnostic capabilities.
**Algorithm 2:** Prostate cancer detection and segmentation framework**Data**: DW-MRI Images**Result**: Segmented Prostate and Lesion Regions, IVIM Parameters, Normalized Dataset, Trained ML Model  **1** **Stage 1: DW-MRI Acquisition**  **2** Acquire DW-MRI images with varying *b*-values  **3** **Stage 2: Preprocessing**  **4** Motion correction, denoising, and image registration  **5** **Stage 3: Prostate and Lesion Segmentation**  **6** Utilize U-Net architecture with Attention U-Net and V-Net  **7** **Stage 4: Extract IVIM Parameters**  **8** Apply IVIM model fitting to obtain S(0), *f*, *D*, and $D^{*}$  **9** **Stage 5: Dataset Normalization****10** Scale and center DW-MRI images and IVIM parameters**11** **Stage 6: ML Learning and Optimization****12** Train ML algorithm on normalized data for prostate cancer detection**13** **Stage 7: Model Evaluation****14** Evaluate the tuned model on a testing dataset**15** Use metrics like balanced accuracy, specificity, sensitivity, and Dice coefficient

## 5. Experiments

The experiments conducted in this study were implemented using the Python programming language on an environment equipped with 256 GB of RAM and a 4 GB NVIDIA GPU. As previously mentioned, the study focused on 80 cases displaying indications of potential prostate cancer.

Segmentation performance assessment experiment: Table 3 presents the results of the suggested segmentation approach, showcasing the achieved accuracy, IoU, and Dice coefficient for evaluation. The results presented in the table provide valuable insights into the performance of different segmentation models and their impact on medical applications. The table displays the accuracy, Dice coefficient, and intersection over union (IoU) metrics achieved by the various models on different regions and modalities. In analyzing the results, we observed that the segmentation models consistently achieved high accuracy across most modalities and regions, indicating their ability to accurately segment the desired ROIs. The Dice coefficient and IoU metrics also demonstrate a high level of agreement between the predicted segmentation and the ground truth, validating the effectiveness of the models. For instance, in the case of the “Axial DWI” modality, all three models (U-Net, Attention U-Net, and V-Net) achieved accuracy levels above 96%, with Dice coefficients and IoU values around 97%. These results highlight the models’ proficiency in accurately segmenting lesions in DWI images, which is crucial for medical diagnosis and treatment planning. Similarly, when examining the “T2-Weighted” modality and “Prostate” region, the U-Net and Attention U-Net models achieved relatively high accuracy levels, while the V-Net model showed lower performance. These results indicate that the U-Net and Attention U-Net models are more effective in accurately segmenting the prostate region in T2-weighted images, which is crucial for prostate cancer diagnosis and treatment planning. The rows highlighted in red were chosen based on their superior performance metrics, with the aim of selecting the most suitable architecture for the segmentation task.

**Figure 6:**
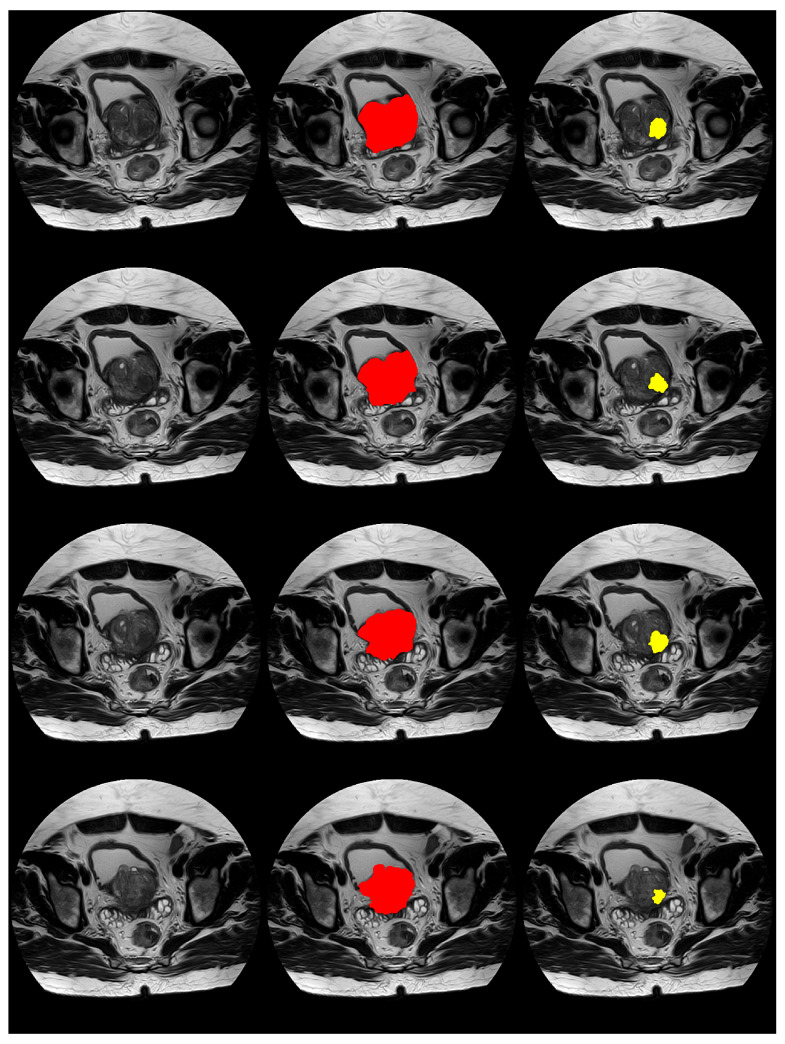
Images displaying visual samples of the segmented sections for a test case. The **left** column represents the scan, the **middle** column represents the prostate, and the **right** column represents the lesion. Each row represents a scan slice.

**Figure 7:**
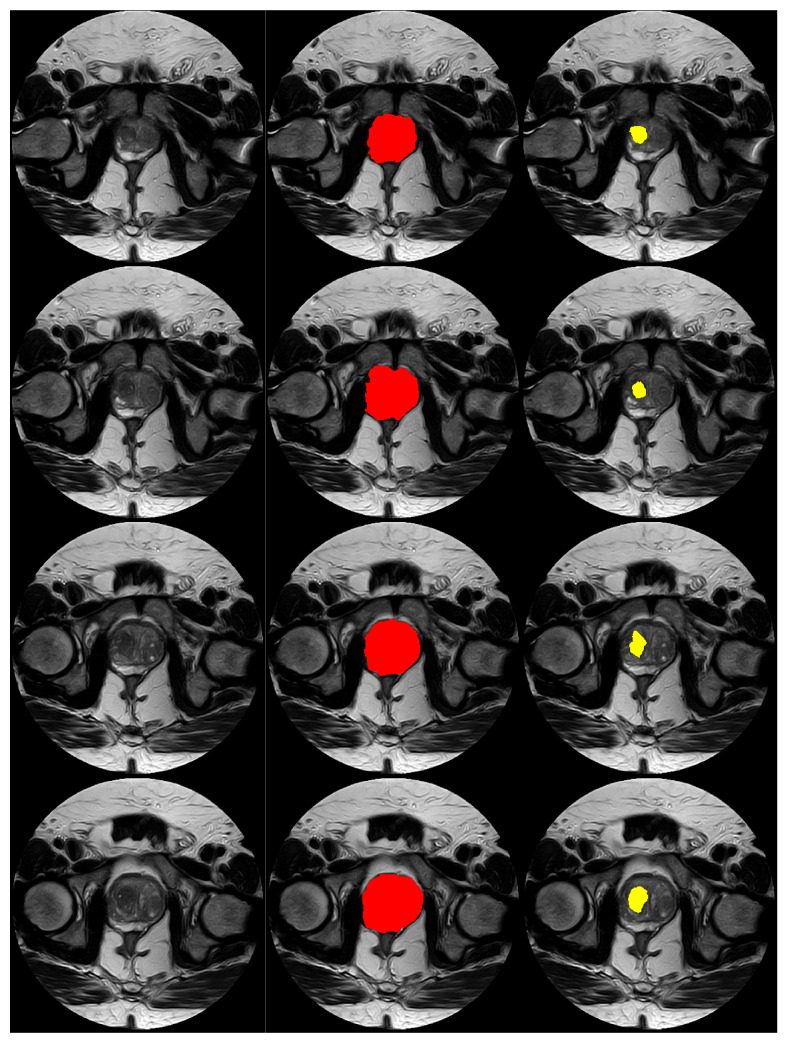
Images displaying visual samples of the segmented sections for another test case. The **left** column represents the scan, the **middle** column represents the prostate, and the **right** column represents the lesion. Each row represents a scan slice.

Further, Figure 6 and Figure 7 display visual examples of the segmented sections for the prostate and lesion of two test cases. In the images, the left column represents the scan, the middle column represents the prostate, and the right column represents the lesion.

Comparison of IVIM parameters with ADC model: In this experiment, the aim was to compare the effectiveness of the bi-exponential model’s IVIM parameters with the mono-exponential ADC model in distinguishing between BPH and PCa. Table 4 compares the performance of different modalities, specifically the IVIM parameters and the ADC model. The evaluation metrics used to assess the performance of each modality included accuracy, sensitivity, specificity, precision, F1 score, ROC curve, and balanced accuracy. Additionally, the table includes information about the classifier, PCA, scaler, and best parameters for each combination of features and modalities.

Looking at the ADC model, it is evident that the multi-layer perceptron (MLP) classifier achieves a mean accuracy of 77.00% with a standard deviation of 5.540. The sensitivity and specificity values are 91.86% and 59.73%, respectively, indicating higher ability to detect true positive samples compared to true negative samples. The precision and F1 score are 73.07% and 81.26%, respectively, indicating a good balance between precision and recall. The area under the ROC curve (AUC) is 77.86%, suggesting a reasonable discrimination ability. The balanced accuracy, which considers both sensitivity and specificity, is 75.80%. The best scaler for this modality is the L1 normalizer, and the best parameters for the MLP classifier are as follows: activation, TanH; hidden layer size, 100; learning rate, adaptive; solver, LBFGS.

For the ADC model with PCA (17 components), the performance improves slightly. The mean accuracy increases to 79.25%, with a lower standard deviation of 2.250. The sensitivity remains high at 87.91%, while the specificity increases to 69.19%. The precision and F1 score improve to 76.87% and 81.99%, respectively. The AUC is 79.13%, the balanced accuracy is 78.55%, and the best parameters for the MLP classifier are as follows: activation, ReLU; hidden layer size, 200; learning rate, adaptive; solver, LBFGS. The inclusion of PCA components enhances the performance by capturing the most relevant information from the data.

Moving on to the prostate zone (PZ), the AdaBoost classifier achieves a mean accuracy of 78.23% with a standard deviation of 1.462. The sensitivity and specificity values are 83.24% and 71.61%, respectively, indicating a balanced ability to detect true positive and true negative samples. The precision and F1 score are 79.50% and 81.31%, respectively, indicating good overall performance. The AUC and balanced accuracy are 77.66% and 77.43%, respectively. The best scaler for this modality is “Robust,” and the best parameters for the AdaBoost classifier are a learning rate 1.0 and 300 estimators.

When using the random forest classifier (RFC) with PCA (6 components) for the PZ, the mean accuracy is slightly lower at 78.00%, with a standard deviation of 1.341. The sensitivity remains high at 85.41%, while the specificity decreases to 68.21%. The precision and F1 score are 78.03% and 81.54%, respectively. The AUC is 77.29%, the balanced accuracy is 76.81%, and the best parameters for the RFC classifier are as follows: class weight, balanced; criterion, Gini; max depth, 15; number of estimators, 200; number of PCA components, 6. Overall, both classifiers perform well for the PZ, with AdaBoost showing slightly better results.

Moving on to the central zone (CZ), the decision tree (DT) classifier without PCA achieves a mean accuracy of 73.82% with a standard deviation of 3.493. The sensitivity is high at 92.30%, while the specificity is considerably lower at 35.83%. The precision and F1 score are 74.79% and 82.60%, respectively. The AUC and balanced accuracy are 70.21% and 64.07%, respectively. The best scaler for this modality is “Robust,” and the best parameters for the DT classifier are the entropy criterion and a maximum depth of 5.

When utilizing the MLP classifier with PCA (4 components) for the CZ, the mean accuracy decreases to 70.64%, with a standard deviation of 3.016. The sensitivity remains high at 82.57%, while the specificity increases to 46.11%. The precision and F1 score are 75.88% and 79.05%, respectively. The AUC and balanced accuracy are 66.93% and 64.34%, respectively. The best scaler for this modality is “Min Max,” and the best parameters for the MLP classifier are as follows: activation, ReLU; hidden layer size, 100; learning rate, adaptive; number of PCA components, 4; solver, LBFGS.

When combining the CZ and PZ features, the RFC classifier achieves a mean accuracy of 84.08% with a standard deviation of 1.144. The sensitivity and specificity values are 93.24% and 71.96%, respectively. The precision and F1 score are 81.48% and 86.96%, respectively, indicating high overall performance. The AUC and balanced accuracy are 83.30% and 82.60%, respectively. The best scaler for this combined modality is “Standardization,” and the best parameters for the RFC classifier are as follows: class weight, none; criterion, Gini; maximum depth, 5; number of estimators, 100.

Finally, when combining the CZ and PZ features with the DT classifier and PCA (8 components), the mean accuracy is 77.54%, with a standard deviation of 2.403. The sensitivity and specificity are 84.59% and 68.21%, respectively. The precision and F1 score are 77.91% and 81.08%, respectively. The AUC and balanced accuracy are 76.88% and 76.40%, respectively. The best parameters for the DT classifier are the entropy criterion, a maximum depth of 5, and eight PCA components.

Overall, the best performance is achieved by the combination of the CZ and PZ features and the RFC classifier, resulting in an accuracy of 84.08% and a balanced accuracy of 82.60%. This combination demonstrates high sensitivity (93.24%) and reasonable specificity (71.96%) along with good precision (81.48%) and F1 score (86.96%). The best scaler for this combination is “Standardization,” and the best parameters for the RFC classifier are as follows: class weight, none; criterion, Gini; max depth, 5; number of estimators, 100.

### Related Studies: Segmentation Comparison

Table 5 provides a comprehensive comparison between our proposed segmentation approach and related studies, showcasing the accuracy, Dice coefficient, and AUC metrics. Our approach stands out by achieving higher accuracy and Dice coefficient values for both prostate and lesion segmentation compared to previous studies. This indicates that our approach provides more accurate and precise segmentation results, which has significant medical implications. In the medical field, accurate segmentation of prostate and lesion regions is crucial for diagnosis, treatment planning, and disease monitoring, particularly for PCa. Precise segmentation enables clinicians to accurately analyze the size, shape, and location of the prostate or the lesions, aiding in treatment decisions and assessment of treatment response over time. Additionally, precise segmentation can help to reduce unnecessary biopsies or surgeries and improve overall patient outcomes.

In the context of prostate classification, the accuracy and AUC of the current approach are reasonable, but are not the highest achieved among all the mentioned studies. Challenges in tumor classification might stem from the intricacies of tumor detection in medical images, which often require specialized techniques and fine-tuning to achieve optimal accuracy. Further optimization and fine-tuning of the machine learning model used in the current approach could potentially improve its classification performance.

Unfortunately, the two studies conducted by Meng et al. [32] and Hu et al. [33] in 2023, while being relevant to lesion segmentation, chose to employ manual segmentation methodologies rather than exploring automatic segmentation approaches. As a result, these studies do not contribute to the evaluation of automatic segmentation techniques as the focus of their investigation.

The current study also introduces an expansion of the segmentation evaluation landscape, with novel results for both prostate and lesion segmentation employing T2-weighted images. Notably, this approach achieves an accuracy of 0.9594 and a Dice coefficient of 0.9626 for prostate segmentation using T2-weighted images, while attaining an even higher accuracy of 0.9789 and a Dice coefficient of 0.9837 for lesion segmentation with the same modality.

The suggested approach stands out, as it achieves higher accuracy and Dice coefficient values for both prostate and lesion segmentation compared to the previous studies. This indicates that the suggested approach provides more accurate and precise segmentation results, which can have significant medical implications. In the medical field, accurate segmentation of prostate and lesion regions plays a crucial role in diagnosis, treatment planning, and monitoring of diseases such as prostate cancer. Accurate segmentation allows clinicians to precisely analyze the size, shape, and location of the prostate or lesions, aiding in treatment decisions and the assessment of treatment response over time. Additionally, precise segmentation can assist in reducing unnecessary biopsies or surgeries and improving overall patient outcomes.

## 6. Discussion

The present study represents a significant advancement in the field of PCa detection and diagnosis by introducing a comprehensive framework that combines DW-MRI with ML techniques. The study’s objectives were successfully met, as evidenced by the developed cascaded framework for PCa detection and segmentation, the utilization of the U-Net architecture for accurate prostate and lesion segmentation, the extraction of IVIM parameters from DW-MRI images, and the comparison of IVIM parameters with the conventional ADC model. These contributions collectively enhance the understanding of noninvasive methods for early detection and diagnosis of PCa, with a particular focus on the CZ and PZ of the prostate. The utilization of the cascaded framework for PCa detection and segmentation represents a novel approach that streamlines the process of identifying and localizing PCa lesions. By integrating multiple stages, including image acquisition, prostate and lesion segmentation, IVIM parameter extraction, dataset normalization, learning and optimization, and model evaluation, the proposed framework ensures systematic and accurate analysis of DW-MRI data. This systematic approach contributes to the reliable identification of PCa lesions and the subsequent enhancement of diagnostic accuracy.

The application of the U-net architecture to prostate and lesion segmentation is a significant advancement in the field of PCa detection. This architecture’s effectiveness in accurately locating and separating PCa lesions within the prostate region underscores the potential of deep learning techniques for improving segmentation precision. The successful segmentation of prostate and lesion regions using the U-net architecture provides a solid foundation for subsequent analyses and contributes to the overall reliability of the proposed framework. Furthermore, the experiments conducted in this study aimed to assess the performance of a proposed segmentation approach for prostate cancer diagnosis and treatment planning. The results presented in Table 3 offer valuable insights into the effectiveness of different segmentation models across various modalities and regions.

In our evaluation of segmentation performance, diverse models consistently achieved remarkable accuracy levels. Notably, in the “Axial DWI” modality, all models attained accuracy surpassing 96%, accompanied by Dice coefficients and IoU values nearing 97%. Additionally, in T2-weighted images, both the U-Net and Attention U-Net models exhibited superior proficiency in precisely delineating the prostate region, which holds critical significance for diagnosis and treatment planning. This superior performance in segmenting the prostate region in T2-weighted images suggests their suitability for prostate cancer diagnosis and treatment planning. Comparing our results with previous studies, it becomes evident that the suggested segmentation approach outperforms several previous investigations in terms of accuracy and spatial overlap. This superiority is especially noteworthy considering the diverse range of segmentation outcomes observed in different studies. The high accuracy and Dice coefficient values achieved by the proposed approach emphasize its potential for improving medical image analysis and enhancing the reliability of diagnosis and treatment decisions.

The extraction of IVIM parameters from DW-MRI images represents a key innovation in this study. The IVIM model’s ability to capture information about tissue microstructure through molecular diffusion and microcirculation processes enriches the diagnostic potential of the proposed framework. The inclusion of IVIM parameters enhances the accuracy of PCa detection and diagnosis by providing valuable insights into tissue characteristics that are not captured by conventional ADC models. This integration of advanced diffusion modeling techniques enhances the framework’s ability to differentiate between benign BPH and PCa, contributing to more accurate diagnostic outcomes.

A thorough comparative analysis between IVIM parameters and the widely-utilized ADC model underscores the superiority of the IVIM-based approach. Employing the MLP classifier yielded a mean accuracy of 77.00%, with sensitivity and specificity values of 91.86% and 59.73%, respectively. The integration of PCA with seventeen components further elevated the accuracy to 79.25%. Particularly noteworthy was the amalgamation of features from the CA and PZ using the RFC, which demonstrated outstanding performance, achieving an accuracy of 84.08% and a balanced accuracy of 82.60%. These results underscore the potential of the proposed methodologies in advancing medical image analysis for prostate cancer diagnosis and treatment planning. Such findings pave the way for enhanced PCa management, offering promising prospects for refining patient care, treatment outcomes, and healthcare efficiency.

Our proposed segmentation approach and the comparative analysis of IVIM parameters and the ADC model offer significant contributions to the field of medical imaging and PCa diagnosis. Accurate segmentation is crucial for precise disease assessment and treatment planning, ultimately leading to improved patient outcomes. The ability to differentiate between BPH and PCa using noninvasive imaging techniques holds considerable promise for early detection and personalized treatment. Overall, the findings presented in this manuscript pave the way for improved medical imaging methodologies and more informed clinical decision-making in PCa management.

## 7. Conclusions and Future Directions

This study significantly advances prostate cancer (PCa) detection and diagnosis through the integration of intravoxel incoherent motion (IVIM) imaging and machine learning. In light of the grave global health implications and economic burdens of PCa, early detection is vital. This study’s novel approach, driven by IVIM imaging and machine learning, has the potential to revolutionize PCa management and improve patient outcomes. Our research introduces a comprehensive framework combining IVIM imaging and machine learning to enhance the accuracy of PCa detection and diagnosis. This framework encompasses various stages, including image acquisition, precise prostate and lesion segmentation, IVIM parameter extraction, dataset normalization, machine learning-based optimization, and model evaluation. This structured methodology ensures precise lesion identification and localization, ultimately improving diagnostic accuracy and treatment planning.

The adoption of U-net architectures for prostate and lesion segmentation represents a significant advancement in PCa detection. The success of the U-net architectures in accurately identifying and segmenting PCa lesions within the prostate demonstrates the potential of artificial intelligence for enhancing segmentation precision. This achievement serves as a strong foundation for further analyses and reinforces the overall credibility of the developed framework. This study’s proposed segmentation approach for PCa diagnosis and treatment planning has yielded impressive outcomes. In the segmentation performance assessment, various models consistently achieved high accuracy levels. This was particularly notable in the “Axial DWI” modality, where all models attained accuracy above 96%, accompanied by Dice coefficients and IoU values around 97%. Moreover, in T2-weighted images, the U-Net and Attention U-Net models demonstrated superior proficiency in accurately delineating the prostate region, which is crucial for diagnosis and treatment planning.

The integration of IVIM parameters into the diagnostic framework represents a pivotal innovation. By capturing microstructural information through molecular diffusion and microcirculation processes, the IVIM model enhances the accuracy of PCa detection and diagnosis. This integration provides insights into tissue characteristics overlooked by conventional ADC models, significantly improving the ability to differentiate between benign BPH and PCa. A compelling comparative analysis between IVIM parameters and the widely-used apparent diffusion coefficient (ADC) model demonstrates the superiority of the IVIM-based approach. The MLP classifier achieved a mean accuracy of 77.00%, with sensitivity and specificity values of 91.86% and 59.73%, respectively. Incorporating PCA with seventeen components enhanced the accuracy to 79.25%. Notably, combining features from the central zone (CZ) and prostate zone (PZ) with the RFC classifier yielded the best performance, boasting an accuracy of 84.08% and a balanced accuracy of 82.60%, highlighting the potential of the proposed methodologies in advancing medical image analysis for prostate cancer diagnosis and treatment planning. These findings pave the way for improved PCa management, demonstrating potential improvements in patient care, treatment outcomes, and healthcare efficiency.

Future directions: While this study represents a pivotal advancement in PCa detection and diagnosis, there remain promising avenues for future exploration. The expansion of the dataset to accommodate a broader diversity of cases and demographics could refine and validate the robustness of the developed framework. Integrating additional imaging modalities such as multiparametric MRI could further enrich the diagnostic power of the CAD system. Advanced feature selection and fusion techniques stand to augment the discriminatory capabilities of the CAD system. Incorporating clinical data, including patient histories and biomarker profiles, could facilitate personalized risk assessment and treatment planning, elevating the CAD system’s clinical utility. The eventual implementation of the proposed IVIM-based CAD system within clinical settings holds substantial promise for enhancing PCa management. Conducting rigorous clinical validation studies is imperative in order to establish the real-world efficacy of the CAD system. Collaborations with medical practitioners and radiologists could provide valuable insights for refining and fine-tuning the CAD system’s performance. As technology continues to evolve, continuous updates to the CAD system could integrate the latest advancements in both IVIM imaging and machine learning techniques, ensuring its relevance and accuracy in an ever-changing medical landscape.

## Figures and Tables

**Table 1:** Summary of related studies on the role of IVIM parameters in PCa detection, sorted by year in ascending order.

Year	Modality	Study	Followed Approach	Limitations (Our Perspective)
1988	DW-MRI	Le-Bihan et al. [17]	Introduced IVIM to distinguish tissue perfusion and diffusion effects, suggesting perfusion could be accurately estimated from DWI alone.	Limited to theoretical proposal; further validation needed through empirical studies.
2015	DW-MRI	Zhang et al. [29]	Demonstrated the superiority of the *D* parameter in Gleason score grading of PCa tumors, highlighting the relevance of *D* and ADC to cell density.	Small sample size; potential bias in patient selection.
2017	DW-MRI	Merisaari et al. [38]	Explored different fitting approaches for IVIM imaging in PCa, showing the monoexponential model outperformed IVIM.	Limited to retrospective analysis; requires prospective validation.
2018	mpMRI	Li et al. [35]	Used SVM classification with image features from mpMRI to classify the GS of PCa within the central gland.	Relatively small dataset; validation on larger cohorts necessary.
2019	Multi-modal	Liu et al. [39]	Introduced a two-phase approach for the complete automation of prostate lesion detection and classification.	Limited to retrospective validation; clinical applicability requires prospective studies.
2020	DW-MRI	Chen et al. [24]	Assessed the relationship between IVIM characteristics and PCa severity, showing potential for distinguishing low-grade from high-grade PCa.	Limited to single-center study; generalizability to diverse populations needs confirmation.
2020	mpMRI	Akamine et al. [36]	Developed an HC model using mp-MRI to differentiate between prostate cancer and normal tissue.	Small patient sample size; validation on larger cohorts essential for robustness.
2022	DW-MRI	Alkadi et al. [37]	Investigated the classification potential of various b-values in DW-MRI for detecting PCa, achieving 90% accuracy.	Relatively small patient cohort; validation on diverse populations necessary.
2022	MRI	Sen et al. [34]	Explored the differentiation ability of quantitative diffusion MRI in various tissue types, effectively differentiating false positive lesions and cancer.	Limited to retrospective analysis; larger prospective studies required for clinical validation.
2022	DW-MRI	Zhang et al. [26]	Explored IVIM’s role in differentiating spinal metastasis and tuberculous spondylitis.	Limited to specific tumor types; generalizability to broader patient populations needs examination.
2023	DW-MRI	Cao et al. [27]	Investigated IVIM’s potential in assessing glioma malignancy and IDH-1 gene type.	Limited to glioma cases; applicability to other tumor types requires investigation.
2023	mpMRI	Meng et al. [32]	Evaluated IVIM’s predictive utility for PSMs and GS upgrading in PCa patients undergoing RP, showing enhanced prediction of PSMs.	Small sample size; validation on larger cohorts necessary for robustness.
2023	MRI	Hu et al. [33]	Investigated the predictive potential of 3D APTw and IVIM imaging for detecting BM in PCa patients, showing superior performance of IVIM.	Limited to bone metastasis cases; validation on broader PCa patient groups needed.

**Table 2:** Hyperparameter configurations for lesion segmentation models: U-Net, Attention U-Net, and V-Net. # means the number of.

	U-NET	Attention U-NET	V-NET
Filter Num	[64, 128, 256, 512, 1024]	[64, 128, 256, 512, 1024]	[64, 128, 256, 512, 1024]
Stack Num Down	2	2	N/A
Stack Num Up	2	2	N/A
Activation	GELU	GELU	GELU
Atten Activation	N/A	ReLU	N/A
Attention	N/A	Add	N/A
Batch Norm	False	True	True
Backbone	VGG19	VGG16	N/A
Pool	False	False	False
Unpool	False	False	False
Freeze Backbone	True	True	N/A
Freeze Batch Norm	True	True	N/A
Weights	N/A	ImageNet	N/A
# Res Ini	N/A	N/A	1
# Res Max	N/A	N/A	3

**Table 3:** Results of the suggested segmentation approach, showcasing the achieved accuracy, IoU, and Dice coefficient for evaluation. The red colored rows reflect the best reported results for each category.

Modality	Region	Model	Loss	Accuracy	Dice	IoU
Axial DWI	Lesion	U-Net	0.112	96.56%	97.19%	97.09%
Axial DWI	Lesion	Attention U-Net	0.109	96.48%	96.44%	96.33%
Axial DWI	Lesion	V-Net	0.082	96.54%	97.26%	97.10%
Axial DWI	Prostate	U-Net	0.319	96.70%	98.29%	98.27%
Axial DWI	Prostate	Attention U-Net	0.292	96.53%	98.19%	98.17%
Axial DWI	Prostate	V-Net	0.119	95.63%	97.14%	97.02%
T2-Weighted	Lesion	U-Net	0.055	96.97%	98.32%	98.20%
T2-Weighted	Lesion	Attention U-Net	0.071	96.97%	98.47%	98.36%
T2-Weighted	Lesion	V-Net	0.051	97.89%	98.37%	98.26%
T2-Weighted	Prostate	U-Net	0.213	93.62%	93.27%	93.14%
T2-Weighted	Prostate	Attention U-Net	0.142	95.94%	96.26%	96.03%
T2-Weighted	Prostate	V-Net	0.160	95.45%	95.13%	95.01%

**Table 4:** Comparison of the performance of different modalities, showing IVIM parameters versus the ADC model. The reported metrics are accuracy, sensitivity, specificity, precision, F1 score, ROC, and balanced accuracy, with mean and standard deviation values reported for each metric. # means the number of.

Features	Classifier	PCA	Accuracy		Sensitivity		Specificity		Precision		F1		ROC		Balanced Accuracy		Scaler	Best Params
			Mean	Std	Mean	Std	Mean	Std	Mean	Std	Mean	Std	Mean	Std	Mean	Std		
ADC	MLP	N/A	77.00%	5.540	91.86%	1.560	59.73%	12.174	73.07%	5.448	81.26%	3.493	77.86%	4.091	75.80%	5.994	L1 Normalizer	Activation: TanH, HLS: 100, LR: Adaptive, Solver: LBFGS
ADC	MLP	17	79.25%	2.250	87.91%	2.712	69.19%	3.461	76.87%	2.155	81.99%	1.943	79.13%	2.235	78.55%	2.283	None	Activation: ReLU, HLS: 200, LR: Adaptive, Solver: LBFGS
PZ	ADA	N/A	78.23%	1.462	83.24%	2.111	71.61%	2.180	79.50%	1.286	81.31%	1.341	77.66%	1.448	77.43%	1.452	Robust	LR: 1.0, # Estimators: 300,
PZ	RFC	6	78.00%	1.341	85.41%	1.576	68.21%	1.557	78.03%	1.022	81.54%	1.172	77.29%	1.331	76.81%	1.335	None	CW: Balanced, Criterion: Gini, MD: 15, # Estimators: 200, # PCA Components: 6
CZ	DT	N/A	73.82%	3.493	92.30%	2.706	35.83%	8.094	74.79%	2.573	82.60%	2.214	70.21%	2.950	64.07%	4.509	Robust	Criterion: Entropy, MD: 5, Splitter: Random
CZ	MLP	4	70.64%	3.016	82.57%	4.207	46.11%	3.557	75.88%	1.509	79.05%	2.508	66.93%	2.827	64.34%	2.699	Min Max	Activation: ReLU, HLS: 100, LR: Adaptive, # PCA Components: 4, Solver: LBFGS
CZ + PZ	RFC	N/A	84.08%	1.144	93.24%	1.351	71.96%	1.964	81.48%	1.071	86.96%	0.930	83.30%	1.134	82.60%	1.192	Standardization	CW: None, Criterion: Gini, MD: 5, # Estimators: 100
CZ + PZ	DT	8	77.54%	2.403	84.59%	2.847	68.21%	3.977	77.91%	2.256	81.08%	2.020	76.88%	2.401	76.40%	2.490	None	Criterion: Entropy, MD: 5, # PCA Components: 8, Splitter: Best

MLP: Multi-Layer Perceptron Classifier; DT: Decision Tree, RFC: Random Forest Classifier; ADA: AdaBoost Classifier; LR: Learning Rate; HLS: Hidden Layer Sizes; CW: Class Weight; MD: Max Depth; Std: Standard Deviation.

**Table 5:** Comparison between the suggested approach and related studies, showcasing the metrics of accuracy, Dice coefficient, and AUC. ↑ means the more the value, the better the metric. ✓ means achieved by the study.

Study	Year	Region	Methodology	Classification	Segmentation	Extracted Parameters	Accuracy ↑	Dice ↑	AUC ↑
Li et al. [35]	2018	Prostate	SVM-Gaussian	✓		IVIM and ADC	-	-	0.91
Liu et al. [39]	2019	Lesion	Mask R-CNN and Weakly Supervised Deep Neural Network	✓	✓	ADC	-	-	0.92
Chen et al. [24]	2020	Lesion	SVM	✓		IVIM	-	-	0.786
Akamine et al. [36]	2020	Prostate	Hierarchical clustering (HC)	✓		IVIM, DKI, and permeability	0.978	-	-
sen et al. [34]	2022	Lesion	Deep Learning	✓		ADC, IVIM, DKI, and VERDICT	-	-	0.9086
Alkadi et al. [37]	2022	Lesion	Generic FeedForward Neural Network	✓		ADC and IVIM	0.9	-	0.978
Meng et al. [32]	2023	Lesion	Logistic regression models		✓	IVIM and clinical characteristics	Manual		
Hu et al. [33]	2023	Lesion	Statistical analysis	✓	✓	3D APTw, DWI, and IVIM	Manual		
Suggested Approach	2024	Prostate (Axial DWI)	U-Net,		✓	IVIM and ADC	0.9670	0.9829	-
		Lesion (Axial DWI)	Attention U-Net,				0.9654	0.9726	-
		Prostate (T2-Weighted)	and V-Net				0.9594	0.9626	-
		Lesion (T2-Weighted)					0.9789	0.9837	-
		Tumor	Machine Learning	✓			0.8408	0.8696	0.8330

## Data Availability

The datasets generated during and/or analyzed during the current study are available from the corresponding author on a reasonable request.

## References

[1] Cancer.Net Prostate Cancer: Introduction. https://www.cancer.net/cancer-types/prostate-cancer/introduction.

[2] McNeal J.E. (1988). Normal histology of the prostate. Am. J. Surg. Pathol..

[3] Balaha H.M., Saif M., Tamer A., Abdelhay E.H. (2022). Hybrid deep learning and genetic algorithms approach (HMB-DLGAHA) for the early ultrasound diagnoses of breast cancer. Neural Comput. Appl..

[4] Marima R., Hull R., Mathabe K., Setlai B., Batra J., Sartor O., Mehrotra R., Dlamini Z. (2021). Prostate cancer racial, socioeconomic, geographic disparities: Targeting the genomic landscape and splicing events in search for diagnostic, prognostic and therapeutic targets. Am. J. Cancer Res..

[5] Deb S., Chin M.Y., Pham S., Adomat H., Hurtado-Coll A., Gleave M.E., Tomlinson Guns E.S. (2021). Steroidogenesis in peripheral and transition zones of human prostate cancer tissue. Int. J. Mol. Sci..

[6] Zhou Z., Zhou Y., Yan W., Sun H., Li Q., Li H., Ji Z. (2021). Unilateral lesion detected on preoperative multiparametric magnetic resonance imaging and MRI/US fusion-guided prostate biopsy is not an appropriate indication for focal therapy in prostate cancer. Urologic Oncology: Seminars and Original Investigations.

[7] Chen J., Guo Y., Guo Y., Jiang M., Zhang Y., Dai Y., Yao X. (2023). Preoperative assessment of microvascular invasion of hepatocellular carcinoma using non-Gaussian diffusion-weighted imaging with a fractional order calculus model: A pilot study. Magn. Reson. Imaging.

[8] He N., Li Z., Li X., Dai W., Peng C., Wu Y., Huang H., Liang J. (2020). Intravoxel incoherent motion diffusion-weighted imaging used to detect prostate cancer and stratify tumor grade: A meta-analysis. Front. Oncol..

[9] Zhang X. (2022). Application of artificial intelligence recognition technology in digital image processing. Wirel. Commun. Mob. Comput..

[10] Balaha H.M., Hassan A.E.S. (2023). A variate brain tumor segmentation, optimization, and recognition framework. Artif. Intell. Rev..

[11] Balaha H.M., Hassan A.E.S. (2023). Skin cancer diagnosis based on deep transfer learning and sparrow search algorithm. Neural Comput. Appl..

[12] Ayyad S.M., Shehata M., Shalaby A., Abou El-Ghar M., Ghazal M., El-Melegy M., Abdel-Hamid N.B., Labib L.M., Ali H.A., El-Baz A. (2021). Role of AI and histopathological images in detecting prostate cancer: A survey. Sensors.

[13] Elgendy M., Shehata M., Alksas A., Ghoneim M., Sherif F., Mahmoud A., Elgarayhi A., Taher F., Sallah M., Ghazal M. (2022). Role of imaging and ai in the evaluation of COVID-19 infection: A comprehensive survey. Front. Biosci. (Landmark Ed.).

[14] Deng H., Cai N., Peng Y. Semi-Quantitative Analysis of DCE-MRI for Classification of the Prostate with and without Cancer. Proceedings of the 2021 6th International Conference on Image, Vision and Computing (ICIVC).

[15] Ayyad S.M., Badawy M.A., Shehata M., Alksas A., Mahmoud A., Abou El-Ghar M., Ghazal M., El-Melegy M., Abdel-Hamid N.B., Labib L.M. (2022). A new framework for precise identification of prostatic adenocarcinoma. Sensors.

[16] Alfano R., Bauman G.S., Gomez J.A., Gaed M., Moussa M., Chin J., Pautler S., Ward A.D. (2022). Prostate cancer classification using radiomics and machine learning on mp-MRI validated using co-registered histology. Eur. J. Radiol..

[17] Le Bihan D., Breton E., Lallemand D., Aubin M., Vignaud J., Laval-Jeantet M. (1988). Separation of diffusion and perfusion in intravoxel incoherent motion MR imaging. Radiology.

[18] Malagi A.V., Netaji A., Kumar V., Baidya Kayal E., Khare K., Das C.J., Calamante F., Mehndiratta A. (2022). IVIM–DKI for differentiation between prostate cancer and benign prostatic hyperplasia: Comparison of 1.5 T vs. 3 T MRI. Magn. Reson. Mater. Phys. Biol. Med..

[19] Catanese A., Malacario F., Cirillo L., Toni F., Zenesini C., Casolino D., Bacci A., Agati R. (2018). Application of intravoxel incoherent motion (IVIM) magnetic resonance imaging in the evaluation of primitive brain tumours. Neuroradiol. J..

[20] Ciritsis A., Boss A., Rossi C. (2018). Automated pixel-wise brain tissue segmentation of diffusion-weighted images via machine learning. NMR Biomed..

[21] Cho G.Y., Moy L., Kim S.G., Baete S.H., Moccaldi M., Babb J.S., Sodickson D.K., Sigmund E.E. (2016). Evaluation of breast cancer using intravoxel incoherent motion (IVIM) histogram analysis: Comparison with malignant status, histological subtype, and molecular prognostic factors. Eur. Radiol..

[22] Almutlaq Z.M., Wilson D.J., Bacon S.E., Sharma N., Stephens S., Dondo T., Buckley D.L. (2022). Evaluation of Monoexponential, Stretched-Exponential and Intravoxel Incoherent Motion MRI Diffusion Models in Early Response Monitoring to Neoadjuvant Chemotherapy in Patients With Breast Cancer—A Preliminary Study. J. Magn. Reson. Imaging.

[23] Meyer H.J., Höhn A.K., Woidacki K., Andric M., Powerski M., Pech M., Surov A. (2021). Associations between IVIM histogram parameters and histopathology in rectal cancer. Magn. Reson. Imaging.

[24] Chen Z., Xue Y., Zhang Z., Li W., Wen M., Zhao Y., Li J., Weng Z., Ye Q. (2020). The performance of intravoxel-incoherent motion diffusion-weighted imaging derived hypoxia for the risk stratification of prostate cancer in peripheral zone. Eur. J. Radiol..

[25] Bao J., Wang X., Hu C., Hou J., Dong F., Guo L. (2017). Differentiation of prostate cancer lesions in the transition zone by diffusion-weighted MRI. Eur. J. Radiol. Open.

[26] Zhang E., Li Y., Xing X., Qin S., Yuan H., Lang N. (2022). Intravoxel incoherent motion to differentiate spinal metastasis: A pilot study. Front. Oncol..

[27] Cao M., Wang X., Liu F., Xue K., Dai Y., Zhou Y. (2023). A three-component multi-b-value diffusion-weighted imaging might be a useful biomarker for detecting microstructural features in gliomas with differences in malignancy and IDH-1 mutation status. Eur. Radiol..

[28] Le Bihan D. (2008). Intravoxel incoherent motion perfusion MR imaging: A wake-up call. Radiology.

[29] Zhang Y.D., Wang Q., Wu C.J., Wang X.N., Zhang J., Liu H., Liu X.S., Shi H.B. (2015). The histogram analysis of diffusion-weighted intravoxel incoherent motion (IVIM) imaging for differentiating the gleason grade of prostate cancer. Eur. Radiol..

[30] Hompland T., Hole K.H., Ragnum H.B., Aarnes E.K., Vlatkovic L., Lie A.K., Patzke S., Brennhovd B., Seierstad T., Lyng H. (2018). Combined MR Imaging of Oxygen Consumption and Supply Reveals Tumor Hypoxia and Aggressiveness in Prostate Cancer PatientsHypoxia Imaging in Prostate Cancer. Cancer Res..

[31] Van Houdt P.J., Yang Y., Van der Heide U.A. (2021). Quantitative magnetic resonance imaging for biological image-guided adaptive radiotherapy. Front. Oncol..

[32] Meng S., Gan W., Chen L., Wang N., Liu A. (2023). Intravoxel incoherent motion predicts positive surgical margins and Gleason score upgrading after radical prostatectomy for prostate cancer. Radiol. Medica.

[33] Hu W., Chen L., Lin L., Wang J., Wang N., Liu A. (2023). Three-dimensional amide proton transfer-weighted and intravoxel incoherent motion imaging for predicting bone metastasis in patients with prostate cancer: A pilot study. Magn. Reson. Imaging.

[34] Sen S., Valindria V., Slator P.J., Pye H., Grey A., Freeman A., Moore C., Whitaker H., Punwani S., Singh S. (2022). Differentiating False Positive Lesions from Clinically Significant Cancer and Normal Prostate Tissue Using VERDICT MRI and Other Diffusion Models. Diagnostics.

[35] Li J., Weng Z., Xu H., Zhang Z., Miao H., Chen W., Liu Z., Zhang X., Wang M., Xu X. (2018). Support Vector Machines (SVM) classification of prostate cancer Gleason score in central gland using multiparametric magnetic resonance images: A cross-validated study. Eur. J. Radiol..

[36] Akamine Y., Ueda Y., Ueno Y., Sofue K., Murakami T., Yoneyama M., Obara M., Van Cauteren M. (2020). Application of hierarchical clustering to multi-parametric MR in prostate: Differentiation of tumor and normal tissue with high accuracy. Magn. Reson. Imaging.

[37] Alkadi R., Abdullah O., Werghi N. (2022). The Classification Power of Classical and Intra-voxel Incoherent Motion (IVIM) Fitting Models of Diffusion-weighted Magnetic Resonance Images: An Experimental Study. J. Digit. Imaging.

[38] Merisaari H., Movahedi P., Perez I.M., Toivonen J., Pesola M., Taimen P., Boström P.J., Pahikkala T., Kiviniemi A., Aronen H.J. (2017). Fitting methods for intravoxel incoherent motion imaging of prostate cancer on region of interest level: Repeatability and gleason score prediction. Magn. Reson. Med..

[39] Liu Z., Jiang W., Lee K.H., Lo Y.L., Ng Y.L., Dou Q., Vardhanabhuti V., Kwok K.W. (2019). A two-stage approach for automated prostate lesion detection and classification with mask R-CNN and weakly supervised deep neural network. Proceedings of the Artificial Intelligence in Radiation Therapy: First International Workshop, AIRT 2019, Held in Conjunction with MICCAI 2019.

[40] Ronneberger O., Fischer P., Brox T. (2015). U-net: Convolutional networks for biomedical image segmentation. Proceedings of the Medical Image Computing and Computer-Assisted Intervention–MICCAI 2015: 18th International Conference.

[41] Oktay O., Schlemper J., Folgoc L.L., Lee M., Heinrich M., Misawa K., Mori K., McDonagh S., Hammerla N.Y., Kainz B. (2018). Attention u-net: Learning where to look for the pancreas. arXiv.

[42] Milletari F., Navab N., Ahmadi S.A. V-net: Fully convolutional neural networks for volumetric medical image segmentation. Proceedings of the 2016 Fourth International Conference on 3D Vision (3DV).

[43] Balaha M.M., El-Kady S., Balaha H.M., Salama M., Emad E., Hassan M., Saafan M.M. (2023). A vision-based deep learning approach for independent-users Arabic sign language interpretation. Multimed. Tools Appl..

[44] Surov A., Meyer H.J., Höhn A.K., Behrmann C., Wienke A., Spielmann R.P., Garnov N. (2017). Correlations between intravoxel incoherent motion (IVIM) parameters and histological findings in rectal cancer: Preliminary results. Oncotarget.

[45] Pang Y., Turkbey B., Bernardo M., Kruecker J., Kadoury S., Merino M.J., Wood B.J., Pinto P.A., Choyke P.L. (2013). Intravoxel incoherent motion MR imaging for prostate cancer: An evaluation of perfusion fraction and diffusion coefficient derived from different b-value combinations. Magn. Reson. Med..

[46] Ranganathan A. (2004). The levenberg-marquardt algorithm. Tutor. Algorithm.

[47] Balaha H.M., Shaban A.O., El-Gendy E.M., Saafan M.M. (2022). A multi-variate heart disease optimization and recognition framework. Neural Comput. Appl..

[48] Yousif N.R., Balaha H.M., Haikal A.Y., El-Gendy E.M. (2023). A generic optimization and learning framework for Parkinson disease via speech and handwritten records. J. Ambient. Intell. Humaniz. Comput..

[49] Baron E. (1996). Classification. Medical Microbiology.

[50] Baghdadi N.A., Alsayed S.K., Malki G.A., Balaha H.M., Farghaly Abdelaliem S.M. (2022). An Analysis of Burnout among Female Nurse Educators in Saudi Arabia Using K-Means Clustering. Eur. J. Investig. Health Psychol. Educ..

[51] Sharaby I., Alksas A., Nashat A., Balaha H.M., Shehata M., Gayhart M., Mahmoud A., Ghazal M., Khalil A., Abouelkheir R.T. (2023). Prediction of wilms’ tumor susceptibility to preoperative chemotherapy using a novel computer-aided prediction system. Diagnostics.

[52] Balaha H.M., Hassan A.E.S. (2023). Comprehensive machine and deep learning analysis of sensor-based human activity recognition. Neural Comput. Appl..

[53] Balaha H.M., Ali H.A., Saraya M., Badawy M. (2021). A new Arabic handwritten character recognition deep learning system (AHCR-DLS). Neural Comput. Appl..

[54] Balaha H.M., El-Gendy E.M., Saafan M.M. (2022). A complete framework for accurate recognition and prognosis of COVID-19 patients based on deep transfer learning and feature classification approach. Artif. Intell. Rev..

[55] Baghdadi N.A., Malki A., Abdelaliem S.F., Balaha H.M., Badawy M., Elhosseini M. (2022). An automated diagnosis and classification of COVID-19 from chest CT images using a transfer learning-based convolutional neural network. Comput. Biol. Med..

[56] Badawy M., Almars A.M., Balaha H.M., Shehata M., Qaraad M., Elhosseini M. (2023). A two-stage renal disease classification based on transfer learning with hyperparameters optimization. Front. Med..

[57] Abdulazeem Y., Balaha H.M., Bahgat W.M., Badawy M. (2021). Human action recognition based on transfer learning approach. IEEE Access.

[58] Balaha H.M., El-Gendy E.M., Saafan M.M. (2021). CovH2SD: A COVID-19 detection approach based on Harris Hawks Optimization and stacked deep learning. Expert Syst. Appl..

[59] Balaha H.M., Antar E.R., Saafan M.M., El-Gendy E.M. (2023). A comprehensive framework towards segmenting and classifying breast cancer patients using deep learning and Aquila optimizer. J. Ambient. Intell. Humaniz. Comput..

[60] Baghdadi N.A., Malki A., Balaha H.M., AbdulAzeem Y., Badawy M., Elhosseini M. (2022). An optimized deep learning approach for suicide detection through Arabic tweets. PeerJ Comput. Sci..

[61] Bahgat W.M., Balaha H.M., AbdulAzeem Y., Badawy M.M. (2021). An optimized transfer learning-based approach for automatic diagnosis of COVID-19 from chest X-ray images. PeerJ Comput. Sci..

